# The Pathogenesis and Therapies of Striated Muscle Laminopathies

**DOI:** 10.3389/fphys.2018.01533

**Published:** 2018-10-30

**Authors:** Astrid Brull, Blanca Morales Rodriguez, Gisèle Bonne, Antoine Muchir, Anne T. Bertrand

**Affiliations:** ^1^Sorbonne Université, INSERM, Institut de Myologie, Center of Research in Myology, UMRS 974, Paris, France; ^2^Sanofi R&D, Chilly Mazarin, France

**Keywords:** Emery-Dreifuss muscular dystrophy (EDMD), A-type lamins, nuclear envelope (NE), muscular dystrophy (MD), cardiomyopathy, emerin, nesprin protein, luma

## Abstract

Emery-Dreifuss muscular dystrophy (EDMD) is a genetic condition characterized by early contractures, skeletal muscle weakness, and cardiomyopathy. During the last 20 years, various genetic approaches led to the identification of causal genes of EDMD and related disorders, all encoding nuclear envelope proteins. By their respective localization either at the inner nuclear membrane or the outer nuclear membrane, these proteins interact with each other and establish a connection between the nucleus and the cytoskeleton. Beside this physical link, these proteins are also involved in mechanotransduction, responding to environmental cues, such as increased tension of the cytoskeleton, by the activation or repression of specific sets of genes. This ability of cells to adapt to environmental conditions is altered in EDMD. Increased knowledge on the pathophysiology of EDMD has led to the development of drug or gene therapies that have been tested on mouse models. This review proposed an overview of the functions played by the different proteins involved in EDMD and related disorders and the current therapeutic approaches tested so far.

## Introduction

Since the first description of a new X-linked muscular disorder by A. Emery, F. Dreifuss and G. Hogan about six decades ago (Dreifuss and Hogan, 1961; Emery and Dreifuss, 1966), Emery-Dreifuss muscular dystrophy (EDMD) is now also described as an autosomal dominant and recessive inherited disease (Chakrabarti and Pearce, 1981; Fenichel et al., 1982; Miller et al., 1985; Serratrice and Pouget, 1986; Witt et al., 1988). In the 1990's, positional cloning analyses led to the identification of two major EDMD causative genes: *EMD* in classical X-linked forms and *LMNA* in autosomal dominant and recessive forms (Bione et al., 1994; Bonne et al., 1999), elucidating around 60% of EDMD cases. These two genes encode proteins of the nuclear envelope, named emerin and A-type lamins, respectively, raising a new research area around the nuclear membranes. More recently, based on the common localization of these proteins, various genetic strategies, including candidate gene approaches, were undertaken in patients suffering from muscular dystrophies without mutations in *EMD* or *LMNA*. This has led to the identification of mutations or variants in genes encoding other nuclear envelope proteins such as FHL1B (Gueneau et al., 2009; Ziat et al., 2016), nesprin 1 and 2 (Zhang et al., 2007), SUN1/2 (Meinke et al., 2014), and LUMA (Liang et al., 2011) associated to a range of muscular dystrophies, some of them sharing clinical features of EDMD. This group of disorders is now collectively named striated muscle laminopathies.

This review focuses on the basal description of: (1) the clinic of striated muscle laminopathies, (2) the functions of the disease-causing proteins, and (3) the different treatment strategies that have been studied so far.

## EDMD, EDMD-like myopathies, and mutated genes

The first EDMD description (Emery and Dreifuss, 1966) concerned a three generation family from Virginia, USA, in which eight males were affected by an unusual type of muscular dystrophy transmitted in an X-linked manner (Dreifuss and Hogan, 1961; Emery and Dreifuss, 1966). It was first considered by McKusick in 1971 as a benign form with contractures of a new X-linked muscular dystrophy (McKusick, 1971) and thereafter included in the Becker's 1972 classification of muscular dystrophies (Becker, 1972).

In its 1986's classification of muscular dystrophies, Becker added the autosomal forms of EDMD to the spectrum of muscular dystrophies known at that time (Becker, 1986). Clinically indistinguishable from the X-linked form, he suggested the Hauptmann-Tannhauser eponym to name these autosomal forms. In view of these various reports, and in agreement with other authors (Serratrice and Pouget, 1986; Witt et al., 1988), Emery suggested to use the term Emery-Dreifuss muscular dystrophy for the disorder characterized by (1) early contractures, (2) humeroperoneal muscle weakness, and (3) cardiac disease characterized by supraventricular arrhythmias, disorders of atrioventricular conduction, and cardiomyopathy (Sanna et al., 2003). Then they were classified as either myopathic or neurogenic in origin, and inherited as an X linked, recessive or autosomal dominant trait (Emery, 1989). This genetic heterogeneity was confirmed thereafter during the 90's and the following decade when the underlying molecular bases of EDMD were identified.

Positional cloning led to the identification of the first molecular defects in X-linked EDMD on chromosome Xq28 in the *STA* gene (now called *EMD*) (Bione et al., 1994), followed a few years later by the identification of a mutation on chromosome 1q21 in the *LMNA* gene (Bonne et al., 1999). These two genes encode proteins of the nuclear envelope (see below), opening a new field of research on striated muscle disorders linked to defects in nuclear envelope proteins. About a decade later, acquired knowledge about the pathophysiology of EDMD has led to the identification of other genes involved in EDMD and EDMD-like disorders. Based on a candidate gene strategy, two different groups identified mutations in the *SYNE1* and/or *SYNE2* genes (Zhang et al., 2007) and missense variants in the *TMEM43* gene (Liang et al., 2011) in the so-called autosomal “EDMD-related myopathy.” Indeed, joint contractures are either lacking or not prominent, and for patients carrying *SYNE1* and/or *SYNE2* mutations, skeletal muscle involvement was highly variable between patients. Finally, a whole-genome analysis of six EDMD families with X-linked inheritance led to the identification of mutations in *FHL1* gene (Gueneau et al., 2009) (Table 1).

**Table 1:** Striated and cardiac muscle laminopathies caused by mutations in nuclear envelope proteins.

***LMNA* Mutations**	
AD-Emery Dreifuss Muscular Dystrophy (AD-EDMD)	Bonne et al., 1999
AR-Emery Dreifuss Muscular Dystrophy (AR-EDMD)	Raffaele Di Barletta et al., 2000
Limb-girdle muscular dystrophy type 1B (LGMD1B)	van der Kooi et al., 1997; Muchir et al., 2000
*LMNA*-associated congenital muscular dystrophy (L-CMD)	Quijano-Roy et al., 2008
Dilated-cardiomyopathy (DCM-CD)	Fatkin et al., 1999
***EMD* Mutations**	
X-linked Emery-Dreifuss Muscular Dystrophy (XL-EDMD)	Bione et al., 1994
X-linked Limb-girdle muscular dystrophy (X-LGMD)	Ura et al., 2007
***LAP2* Mutations**	
*LAP-2α*: Dilated cardiomyopathy (DCM)	Taylor et al., 2005
***SYNE* Mutations**	
Nesprin 1α and 2β: Emery-Dreifuss Muscular Dystrophy-like	Zhang et al., 2007
Nesprin 1α: Dilated cardiomyopathy	Puckelwartz et al., 2010
***TMEM43* Mutations**	
Emery-Dreifuss Muscular Dystrophy-like	Liang et al., 2011
***FHL1* Mutations**	
Emery-Dreifuss Muscular Dystrophy	Gueneau et al., 2009

## Proteins involved in EDMD

**Figure 1:**
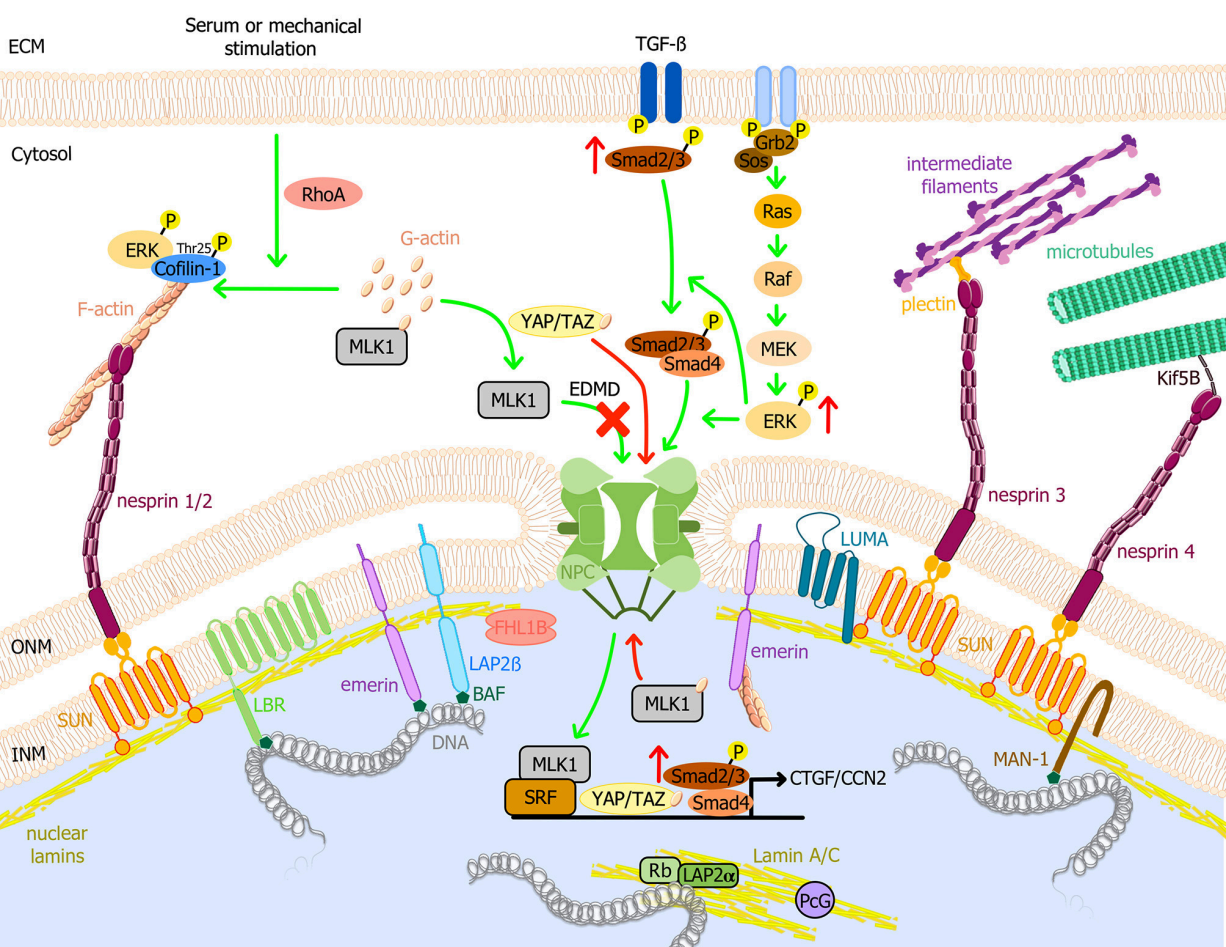
Schematic model of the nuclear envelope proteins and their potential roles in EDMD physiopathology. Nuclear lamins form a meshwork underneath the INM connected with the cytoplasm. It interacts with TM proteins of the nuclear envelope, i.e., emerin, LBR, LAP2, SUN1/2, and MAN1, and with several transcription factors such as Rb. Through the LINC complex, A-type lamins interact with actin microfilaments, microtubules, and cytoplasmic intermediate filaments, connecting the nuclear lamina to the extracellular matrix. MAPK pathways are important transduction cascades initiated by extracellular mitogens, growth factors and cytokines at the cell surface and finalized to the nucleus to control gene expression, regulating cell proliferation and differentiation, survival and apoptosis. *LMNA* mutations causing EDMD have been related to the activation of ERK, JNK, and p38α cascades, leading to the posterior activation of CTGF/CCN2 via TGF-ß/Smad signaling pathway and cofilin-1, the latter leading to actin depolymerization. After serum or mechanical stimulation, RhoA promotes cytoplasmic actin polymerization, causing the release of MKL1 from cytoplasmic G-actin. MKL1 translocates into the nucleus and together with SRF induces gene expression. In addition, emerin facilitates polymerization of nuclear actin, reducing the nuclear export of MKL1 to the cytoplasm. In EDMD cells, emerin mislocalizes and is unable to modulate nuclear actin polymerization. G-actin binds to MKL1 and it is exported from the nucleus, impairing gene expression. YAP and TAZ are key transcription factors for cell proliferation. YAP/TAZ activation causes their nuclear accumulation, promoting cell proliferation, and inhibiting differentiation. Nuclear localization of YAP/TAZ is increased in patient myoblasts with *LMNA* mutations. Green arrows indicate reported pathways and red arrows indicate altered EDMD reported pathways. BAF, barrier-to-autointegration factor; ECM, Extracellular matrix; EDMD, Emery-Dreifuss muscular dystrophy; ERK, extracellular signal-regulated kinase; F-actin, actin filament; G-actin, globular actin; Grb2, growth factor receptor-bound protein 2; INM, inner nuclear membrane; Kif5B, kinesin family member 5B; LAP, lamin associated protein; LBR, lamin B receptor; MAPK, mitogen-activated protein kinase; MKL1, megakaryoblastic leukemia 1; NPC, nuclear pore complex; ONM, outer nuclear membrane; Raf, proto-oncogen serine/threonine-protein kinase; Rb, retinoblastoma protein; RhoA, ras homolog family member A; Sos, son of sevenless homolog 1; SRF, serum response factor; TGF-ß, transforming growth factor ß; YAP, yes-associated protein 1; TAZ, transcriptional coactivator with PDZ-binding motif.

The nuclear envelope is a lipid bilayer membrane that separates the cytoplasm from the nucleus in eukaryotic cells and that encloses the genetic material (Watson, 1955; Aaronson and Blobel, 1975). The nuclear envelope is composed of the inner nuclear membrane (INM) and the outer nuclear membrane (ONM) (Figure 1). The ONM is continuous with the rough endoplasmic reticulum membrane sharing many of its protein content, with the exception of some integral proteins that are retained at the ONM through specific interactions with INM proteins (like nesprins at the ONM that interact with SUN at the INM, both composing the linker of the nucleoskeleton and the cytoskeleton (LINC) complex) (Crisp et al., 2006). By contrast, the INM contains its own integral proteins, like LEM-domain proteins (LAP2, Emerin, MAN1) or LUMA. INM and ONM interact at the site of nuclear pores and through the LINC complex (Crisp et al., 2006; Starr and Fridolfsson, 2010; Luxton and Starr, 2014). Underneath the INM, the nuclear lamina, a protein meshwork composed of A- and B-type lamins, is connected to the cytoskeleton via the LINC complex. The nuclear lamina is involved in different nuclear functions such as DNA replication and chromatin organization but also has important roles in cytoplasmic organization and cytoskeletal mechanotransduction (Lammerding et al., 2004; Hale et al., 2008; Lombardi et al., 2011).

### Lamins

Lamins are type V intermediate filament proteins (Fisher et al., 1986; Goldman et al., 1986; McKeon et al., 1986). As all intermediate filament proteins, lamins possess an α-helical coiled-coil central rod domain, composed of heptad repeats of amino acids, bordered by a globular amino-terminal “head” domain and a carboxyl-terminal “tail” domain. This latter also comprises an Ig-like domain and a nuclear localization sequence (NLS) (Parry et al., 1986; Frangioni and Neel, 1993; Dhe-Paganon et al., 2002; Krimm et al., 2002), which make lamins the only intermediate filament protein found in the nucleus. Based on their sequences and structural properties, nuclear lamins can be separated in A-type and B-type lamins (Gerace et al., 1978). In mammals, three genes encode for lamins: *LMNA, LMNB1*, and *LMNB*2. *LMNA* encodes for A-type lamins, with lamins A and C being the main isoforms generated by alternative splicing and expressed in all differentiated cells (Lin and Worman, 1993; Dechat et al., 2010). Lamins AΔ10 and C2 represent minor isoforms specifically expressed in tumoral cells and in male germ line, respectively (Furukawa et al., 1994; Machiels et al., 1996; Alsheimer et al., 2000). Lamins A and C are identical for their first 566 amino acids and vary in their carboxyl terminal tails. The most abundant B-type lamins are lamin B1, encoded by *LMNB1*, and lamin B2 encoded by *LMNB2*, both constitutively expressed (Gruenbaum et al., 2005; Burke and Stewart, 2013).

Lamin A and B-type lamins are synthesized as precursors that are post-translationally processed. Firstly a farnesylation of the cysteine of the C-terminal CaaX motif of prelamin A, lamin B1 and B2 occurs, leading to the anchorage of lamins in the nuclear membrane (Farnsworth et al., 1989; Beck et al., 1990). Secondly the aaX amino acids are cleaved by endopeptidases (either Rce1 or Zmpste24) (Nigg et al., 1992; Varela et al., 2005; Young et al., 2005). Thirdly the cysteine residue is methylated by Icmt1 (Maske et al., 2003). Finally, lamin A, but not lamin B1 and B2, is subjected to a last post-transcriptional modification consisting in the cleavage of the last 15 residues containing the farnesylated residue, resulting in mature lamin A, which is release from the INM.

Another piece of complexity in lamins arises from their polymerization. Lamins form coiled-coil homo-dimers that further interact longitudinally through head-to-tail association to form a long polar 10 nm polymer that can further assemble laterally (Aebi et al., 1986; Ben-Harush et al., 2009). Recent studies are however challenging this view and described lamins as being 3.5 nm filaments (Harapin et al., 2015; Turgay et al., 2017). Finally, the different types of lamins assemble in separated networks with B-type lamin network lying close to the nuclear envelope while the A-type lamin network lies closer to the nucleoplasm (Shimi et al., 2015; Xie et al., 2016).

### Emerin

The *EMD* gene encodes for an ubiquitous type II TM protein called emerin, anchored to the INM through its C-terminal hydrophobic domain (Manilal et al., 1996; Nagano et al., 1996; Tsuchiya et al., 1999). In the nucleoplasm, emerin interacts with numerous proteins, mainly via its LEM domain. It also interacts with lamin A/C, nesprin-1α and LUMA (Ellis, 2006; Liang et al., 2011). Emerin is able to self-assemble *in vitro* and it was suggested that the LEM domain configuration in the emerin multimers may modulate its interaction with chromatin and nucleoplasmic proteins (Berk et al., 2014; Samson et al., 2017). Depending on tissue, emerin was also described at alternative subcellular locations. This includes endoplasmic or sarcoplasmic reticulum, nucleoplasm, ONM, plasma membrane, and centrosome (Cartegni et al., 1997; Ostlund et al., 1999; Lattanzi et al., 2000; Squarzoni et al., 2000; Manta et al., 2004; Salpingidou et al., 2007). Wheeler et al. proposed that cell type-specific signaling events, such as emerin phosphorylation, might be responsible for targeting emerin to alternative cellular localizations (Wheeler et al., 2010). Emerin functions include regulation of transcription factor localization and function, cell signaling, mechanotransduction, nuclear envelope structure as well as chromatin organization (Koch and Holaska, 2014).

### Nesprins

Six different genes named *SYNE-1, SYNE-2, SYNE-3, SYNE-4, KASH5*, and *LRMP* encode for multiple spliced isoforms of nesprins (for nuclear envelope spectrin repeat proteins) (Zhou et al., 2018). While nesprin-4, KASH5, and LRMP expression is restricted to epithelial cells, meiotic cells, and a subset of taste receptor cells respectively; nesprin-1, −2, and −3 are ubiquitously expressed with a peculiar abundance of nesprin-1 and−2 in striated muscles. Numerous initiation codons and alternative splicing of *SYNE-1* and *SYNE-2* genes lead to a wide variety of protein isoforms with great variability in their molecular weight. Some of these smaller isoforms are restricted to specific tissues, like nesprin-1α2 and nesprin-2α1 for striated muscle (Holt et al., 2016).

Giant nesprin-1 and −2 are characterized by a C-terminal Klarsicht/ANC-1/Syne homology (KASH) domain that anchors the proteins to the nuclear envelope, an N-terminal Calponin Homology (CH) domain involved in interactions with actin, and a central domain constituted of multiple spectrin repeats (Rajgor and Shanahan, 2013). Beside interactions with actin, recent data have shown that they are also connected to microtubules through interactions with motor protein kinesin-1 and dynein/dynactin (Wu et al., 2018). Nesprin-3, that lacks the N-terminal actin-binding domain, is involved in the interaction with intermediate filaments through plectin (Ketema and Sonnenberg, 2011). Shorter isoforms may lack some of these domains. While nesprin-1 and−2 giant are set at the ONM where they interact with SUN proteins, the shorter isoforms like nesprin-1α can also be found at the INM and interact directly with lamin A/C and emerin (Mislow et al., 2002).

### SUN

SUN (Sad1/UNC-84) proteins were named after the identification of their homolog in yeast (Sad1) and *C. elegans* (UNC-84) (Hagan and Yanagida, 1995; Malone et al., 1999). In mammals, five SUN proteins have been described, SUN1, SUN2, SUN3, SUN4, and SUN5. SUN 1 and SUN 2 are ubiquitously expressed, while other isoforms are only expressed in testes (Sosa et al., 2013).

SUN1 and SUN2 comprise a nucleoplasmic N-terminal region that interacts with lamins (Crisp et al., 2006), a single TM domain that spans the INM, followed by a coiled-coil region that protrude in the perinuclear space, and required for the trimerization of SUN proteins (Sosa et al., 2012; Wang et al., 2012; Zhou et al., 2012; Hennen et al., 2018; Jahed et al., 2018). Finally, the C-terminal SUN domain interacts with the KASH domain of nesprins (Sosa et al., 2012; Wang et al., 2012; Zhou et al., 2012).

The nuclear bridge formed between nesprins and SUN proteins (LINC complex) connect the cytoskeleton (actin, microtubules, and intermediate filaments depending on the nesprin isoform) to the nucleoskeleton (lamin A/C and emerin). It provides structural support to the nucleus and plays an essential role in regulating gene expression and mechanotransduction (Wang et al., 2009; Lombardi et al., 2011).

### LUMA

LUMA (or TMEM43) was first identified in a proteomics screen for nuclear envelope proteins (Dreger et al., 2001). It is characterized by four TM domains involved in its oligomerization and a large fragment protruding in the lumen of the endoplasmic reticulum and the perinuclear space (Bengtsson and Otto, 2008). At the INM, it interacts with lamins A/C and B1 as well as with emerin and SUN2 (Bengtsson and Otto, 2008; Liang et al., 2011). The localization of LUMA at the INM depends on A-type lamins while depletion of LUMA induces emerin mislocalization in HeLa cells but not in mouse neonatal cardiomyocytes (Bengtsson and Otto, 2008; Stroud et al., 2018). In the heart, LUMA is found in the intercalated disks where it has been described as a component of the composite junction (Franke et al., 2014; Siragam et al., 2014). At this specific location, LUMA is supposed to participate in the organization and function of proteins involved in cardiac conduction (Siragam et al., 2014).

### FHL1

The *FHL1* gene, localized on the X chromosome, is composed of 8 exons encoding various isoforms of FHL1 (for Four-and-a-Half LIM domain) (Greene et al., 1999). LIM domains (acronym for Lin-11, Isl-1 and Mec3) are highly conserved sequences comprising two zinc fingers in tandem, which are implicated in numerous interactions, essentially with other proteins (Kadrmas and Beckerle, 2004). Each zinc finger is composed of four highly conserved cysteine or histidine linking together one zinc ion (Michelsen et al., 1993; Kosa et al., 1994).

The two first exons are non-coding while exons 6 and 7 are alternatively spliced, which results in three different transcripts: FHL1A, FHL1B and FHL1C; FHL1A being the major isoform in striated muscles. FHL1A is constituted of four and a half LIM domains involved in multiple protein interactions (Lee et al., 1998; Brown et al., 1999a; Greene et al., 1999). FHL1B is composed of only three and a half LIM domain in its N-terminal part, followed by three NLS and one NES, and a binding domain to the RBP-J transcription factor (Brown et al., 1999b). FHL1C is composed of only two and a half LIM domains directly followed by the RBP-J binding domain (Brown et al., 1999b; Ng et al., 2001). Recent data have shown that FHL1B is accumulated at the nuclear lamina where it co-localizes with lamin A/C or emerin (Ziat et al., 2016).

Beside this location at the nuclear envelope, the various FHL1 isoforms have been reported at different locations within the cell: at the focal adhesions where it regulates cell migration via integrins (Robinson et al., 2003); in the sarcomeres where it interacts with MyBP-c as well as with titin and it is involved in sarcomere formation and in the stress-response signaling via activation of the MAPK signaling (McGrath et al., 2006; Sheikh et al., 2008); and in the nucleus where it interacts with various transcriptional factors (like NFAT proteins or RBP-J) involved in cell proliferation and differentiation but also with the pro-apoptotic protein Siva where it is involved in cell survival (Taniguchi et al., 1998; Schulz and Yutzey, 2004; Liang et al., 2008; Cottle et al., 2009).

## Function of the proteins and related pathomechanisms leading to EDMD and related disorders

The use of patient cells and the development of animal models have helped understanding the pathophysiology of EDMD and related disorders, and more specifically, how ubiquitous proteins give rise to striated muscle specific defects. It is now clear that the different proteins involved in EDMD serve as sensors of mechanical tension of the cytoskeleton leading to an adaptive response of the cell via the regulation of the transcription.

### Link with the cytoskeleton

EDMD proteins have been implicated in nuclear positioning, nuclear shape and mechanosensing via their interactions with the cytoskeleton (Chang et al., 2015). All cytoskeleton networks (actin, microtubule and intermediate filaments) have been shown to be disorganized in *Lmna*^Δ8−11^ mouse embryonic fibroblasts (MEF), in 3T3 *Lmna* knock-down cells and in various mouse models (Nikolova et al., 2004; Houben et al., 2009; Chatzifrangkeskou et al., 2018). Experiments performed on MEF of *Lmna*^Δ^^8−11^ or *Emd*-KO mice have shown a disconnection of the nucleus from the rest of cells (Lammerding et al., 2004, 2005; Zwerger et al., 2013) evidenced by the increased centrosome-nucleus distance (Lee et al., 2007; Hale et al., 2008). Shortly after, it was shown that nesprin-2 was a major player in the connection between the nuclear lamina (via interaction with SUN proteins on one side), and the microtubule network (via connection with kinesin light chain-1 on the other side) (Schneider et al., 2011). Cells with disconnection between the cytoplasm and the nucleus have impaired cell polarity, adhesion and migration, which finally impact on myoblasts fusion (Lee et al., 2007; Hale et al., 2008; Chang et al., 2015). In cardiomyocytes, nesprin-2 compensates the loss of nesprin-1, and only nesprin-1/2 double KO mice develop cardiomyopathy (Banerjee et al., 2014).

As mentioned earlier, differentiating myoblasts express specific nesprin isoforms (Randles et al., 2010; Holt et al., 2016). Using various mouse models of mutant nesprin-1 (Table 2), the major roles played by nesprin-1α in nuclear morphology and positioning in skeletal muscles were demonstrated (Zhang et al., 2007, 2010; Puckelwartz et al., 2009; Stroud et al., 2017). Further analyses performed on cultured myoblasts have shown that nesprin-1α isoform is required during muscle differentiation to reorganize the microtubule network from the centrosome in myoblasts to the ONM in myotubes (Gimpel et al., 2017). This reorganization is a prerequisite for the correct nuclear positioning in muscle cells, and is perturbed in C2C12 that are knocked-down for nesprin-1α, in several mouse models, and in patient myoblasts with specific nesprin-1α deletion (Gimpel et al., 2017).

**Table 2:** List of EDMD and EDMD-like myopathies mouse models.

**Model**		**Features**	**References**
***Lamin A/C***			
KO	*Lmna*^−/−^ (*Lmna^Δ^*^8−11^)	EDMD and DCM-CD	Sullivan et al., 1999; Jahn et al., 2012
KO	*Lmna*^GT−/−^	Impaired post-natal cardiomyocyte hypertrophy, skeletal muscle hypotrophy and metabolic defects	Kubben et al., 2011
cKO	*Lmna*^Δ/Δ^	Similar to *Lmna*^GT−/−^ mice with several growth retardation and postnatal lethality at P16-18 days	Kim and Zheng, 2013
cKO	*Lmna-*Z*p*3	Reduced transcriptional activation of muscle related genes in isolated muscle progenitor cells.	Solovei et al., 2013
KI	*Lmna*^H222P/H222P^	AD-EDMD and DCM-CD	Arimura et al., 2005
KI	*Lmna*^N195K/N195K^	DCM-CD	Mounkes et al., 2005
Tg	*Lmna*^M371K/M371K^	EDMD and DCM	Wang et al., 2006
KI	*Lmna*^Δ*K*32/Δ*K*32^	L-CMD, defective skeletal and cardiac muscles maturation and metabolic defects	Bertrand et al., 2012
KI	*Lmna*^Δ*K*32/+^	DCM	Cattin et al., 2013
KI	*Lmna^*PLAO*^*	No defects	Coffinier et al., 2010
KI	*Lmna^*LAO*^*	No defects	Coffinier et al., 2010
KI	*Lmna^*LCO*^*	No defects	Fong et al., 2006
***Emerin***			
KO	*Emd* ^−/−^	Muscle regeneration defects	Melcon et al., 2006
KO	*Emd* ^−/−^	Motor coordination abnormalities, atrio-ventricular conduction defects	Ozawa et al., 2006
**Nesprins**			
KI	Nesprin 1^Δ**KASH**^	EDMD and DCM-CD	Puckelwartz et al., 2009, 2010
KI	Nesprin 1^rKASH^	Increase in centralized myonuclei and displacement of synaptic myonuclei	Zhang et al., 2007
KO	Nesprin-1^−/−^	Increase in centralized myonuclei and myonuclei clustering	Zhang et al., 2010
KI	Nesprin-2^rKASH^	No defects	Zhang et al., 2007
Tg	MCK-KASH^Nesprin−2^	Displacement of synaptic myonuclei.	Zhang et al., 2007
dKO	Nesprin-1^−/−^/Nesprin-2^−/−^	Neonatal lethality with respiratory defects	Zhang et al., 2007
cdKO	Nesprin 1^f/f^/Nesprin 2^−/−^/Nkx2.5Cre	Nuclear elongation and cardiomyopathy with fibrosis and apoptosis	Banerjee et al., 2014
KO	Nesprin 1^Δ*CH*^	No striated muscle defects	Stroud et al., 2017
KO	Nesprin 1α2^−/−^	Centralization and clustering of myonuclei and Kinesin-1 displacement from the NE	Stroud et al., 2017
***SUN***			
KO	Sun1^−/−^	Impairment of telomere attachment to the nuclear envelope, persistent double-strand breaks, inefficient homologous pairing and synapsis formation in meiosis and reproductively infertile	Ding et al., 2007
KO	Sun2^−/−^	No defects	Lei et al., 2009
dKO	Sun1^−/−^/Sun2^−/−^	Neonatal lethality and disruption organization of non-synaptic nuclei	Lei et al., 2009
***LUMA***			
KO	*Luma*^−/−^	No defects	Stroud et al., 2018
KI	*Luma*^S358L/S358L^	No defects	Stroud et al., 2018
***FHL1***			
KO	*Fhl1*^−/−^	Blunted response of heart to pressure overload and other hypertrophic stimuli and decreased MAPK activation	Sheikh et al., 2008
Tg	Tg-*Fhl1*	Increased muscle fiber size, myoblast fusion and NFATc1 activation	Cowling et al., 2008
KO	*Fhl1*-null	Early muscle fiber differentiation and maturation defects. Myofibrillar and intermyofibrillar disorganization, impaired force production and muscle fatigue	Domenighetti et al., 2014

*cKO, conditional knock-out; cdKO, conditional double knock-out; KI, knock-in; KO, knock-out; dKO, double knock-out; Tg, Transgenic*.

Alterations of the different cytoskeleton networks have great impact on striated muscle function. This has been more widely explored in the heart, where *Lmn*a^Δ8−11/Δ8−11^ cardiomyocytes show progressive disorganization and detachment of desmin from the nuclear membrane that parallels the cardiac contractile dysfunction (Nikolova et al., 2004). In *Lmna*^H222P/H222P^ hearts (Table 2), it was shown that microtubule cytoskeleton alteration and decreased acetylation of α-tubulin lead to connexin-43 displacement from the gap-junction, ultimately leading to electrical conduction disturbances (Le Dour et al., 2017; Macquart et al., 2018).

Cytoskeleton components and adhesion complexes are known mechanosensitive elements able to activate numerous signaling pathways such as mitogen-activated protein kinase-extracellular signal-regulated kinase (MAPK-ERK) and to induce the nuclear translocation of MKL1 (also known as MAL or MRTF-A) and YAP/TAZ to activate expression of early responsive genes. Interestingly, ERK pathway has been shown to be activated in the heart of *Lmna*^H222P/H222P^, *Lmna*^Δ^^8−11/Δ8−11^, and *Emd*^−/y^ mice at baseline while it was not in *Fhl1*^−/y^ hearts even after cardiac pressure overload (Muchir et al., 2007a,b; Sheikh et al., 2008; Frock et al., 2012). In muscle cells, while a transient activation of ERK is required few minutes after switching C2C12 myoblasts in differentiation medium, C2C12 where emerin has been knock-down have an increased and prolonged activation of ERK, leading to delayed myotube formation (Huber et al., 2009). Nuclear translocation of MKL1 was inefficient in *Lmna*^Δ^^8−11/Δ8−11^, *Emd*^−/y^, and *Lmna*^N195K/N195K^ MEF as well as in patient skin fibroblasts with *LMNA* mutation leading to isolated cardiomyopathy (*LMNA* p.K219T) following serum stimulation, while it was not altered in another patient's skin fibroblasts with *LMNA* mutations leading to congenital muscular dystrophy (*LMNA* p.K32del) (Ho et al., 2013a,b). Finally, mRNA of the YAP/TAZ pathway were increased in *Emd*^−/y^ myoblasts, while at the protein level the nuclear localization of YAP/TAZ was increased in patient myoblasts with *LMNA* mutations cultured at baseline compared with WT, but not following mechanical stimulation or increase matrix stiffness (Bertrand et al., 2014; Iyer et al., 2017).

In addition, an increasing number of studies are now focusing on the role of the nucleus itself as a mechanosensitive element, mainly through the LINC complex (Kirby and Lammerding, 2018). Analyses made on isolated nuclei have shown that force applied on nesprin-1 is responsible for nuclear stiffening due to a rapid phosphorylation of emerin on Tyr74 and Tyr95, leading to the local recruitment of lamin A/C (Guilluy et al., 2014). Knock-down of lamin A/C, double knock-down of SUN1 and SUN2 or phosphoresistant 74-95FF emerin mutant cells all failed to respond to the stimuli. Absence of mechanotransduction due to mutations or deletion of EDMD proteins is evidenced by the absence of hypertrophy following cardiac pressure overload in *Lmna*^Δ^^8−11/+^, *Fhl1*^−/y^ or nesprin-1/nespin-2 double knock-out mice (Sheikh et al., 2008; Cupesi et al., 2010; Banerjee et al., 2014). Emerin's function in mechanotransduction might be slightly different as *Emd*^−/y^ mice subjected to cardiac pressure overload present with normal cardiac hypertrophy, however they still display impaired cardiac function compared to WT (Stubenvoll et al., 2015).

Activation of these pathways may affect downstream processes leading to cardiomyopathy and muscle weakness observed in EDMD. MKL1 activation leads to cardiac fibrosis following myocardial infarction or angiotensin II stimulation in WT heart (Small et al., 2010). Similarly ERK activation is able to induce cardiac fibrosis via activation of CTGF/CCN2 in heart from *Lmna*^H222P/H222P^ mice (Chatzifrangkeskou et al., 2016). ERK1/2 was also shown to catalyze phosphorylation of cofilin-1 in heart from *Lmna*^H222P/H222P^ mice leading to actin depolymerisation (Figure 1) (Chatzifrangkeskou et al., 2018). At the opposite, YAP/TAZ-dependent activation of FHOD1 observed in patient myoblasts with mutations in *LMNA* or *SYNE*-1 leads to actin polymerization (Bertrand et al., 2014; Schwartz et al., 2017).

### Link with the chromatin and gene regulation

As shown by the specific distribution of chromatin and chromosomes in the nucleus, genome organization is not random (Croft et al., 1999) and depends on tissue; e.g., fibroblasts tend to have an uniform distribution of heterochromatin throughout the periphery while epithelial cells present a more patchy distribution (Zuleger et al., 2011). The patterns of chromatin distribution and chromosome positioning are disrupted in cells from patients with nuclear envelope diseases (Sewry et al., 2001; Maraldi et al., 2006), suggesting an important functional role of the spatial genome organization.

Nuclear pore complex proteins, lamins, and several nuclear envelope transmembrane proteins of the INM interact directly with DNA, chromatin and/or chromatin-associated proteins (Mattout-Drubezki and Gruenbaum, 2003). Chromatin contacts with A- or B-type lamins via specific genomic regions called lamina-associated domains (LADs). LADs are gene-poor, AT-rich and heterochromatic regions that dynamically bind to and dissociate from the nuclear lamina at the nuclear periphery (Guelen et al., 2008; Kind et al., 2013; Lund et al., 2015). Other INM proteins such as LEM-domain proteins indirectly interact with chromatin via the barrier-to-autointegration factor protein (BAF) in a highly ordered nucleoprotein complex (Figure 1) (Furukawa, 1999; Zheng et al., 2000; Lee et al., 2001; Mansharamani and Wilson, 2005). Through interactions with BAF, LEM-domain proteins contribute to the tethering of genomic regions to nuclear periphery, connecting interphase chromosomes to the nuclear lamina, thereby intervening in global nuclear organization (Barton et al., 2015).

Transmission of external forces from the extracellular matrix into the nucleus via the LINC complex (Lombardi et al., 2011) causes deformation of the nuclear envelope and lamina, which, in turn, modulates chromatin organization and transcriptional activity (Swift et al., 2013). Paulsen and colleagues showed that the overexpression of Flag-tagged wild-type lamin A/C, the constantly farnesylated mutant p.L647R or lamin A/C p.R388P (causing congenital muscular dystrophy), resulted in different lamin A/C-genome interaction patterns detectable by 3D genome modeling (Chrom3D) from Hi-C and CHIP-seq data, suggesting that laminopathy-causing mutations can also alter interactions with chromatin (Paulsen et al., 2017). Recent studies have evidenced the participation of nuclear envelope proteins regulated in a cell type-specific manner by reversible sequestration of regulatory elements at the nuclear periphery, leading to their repression (Lund et al., 2013; Ronningen et al., 2015; Robson et al., 2016). In link with this observation, Favreau and colleagues showed that the overexpression of lamin A/C p.R453W (the mutation's hot spot for *LMNA* in EDMD) in C2C12 myoblasts resulted in impaired differentiation to myotubes (Favreau et al., 2004). In addition, PARP1 and CTCF, both altered in *Lmna*^H222P/H222P^ hearts (Chatzifrangkeskou et al., 2016; Vignier et al., 2018) regulate circadian transcriptional plasticity, mediating chromatin interactions, and promoting the recruitment of circadian genes to the nuclear periphery (Zhao et al., 2015). Whereas lamin B1 is localized in the nuclear periphery and interacts only with heterochromatin LADs, lamin A/C binds to both hetero- and euchromatin (Gesson et al., 2016). Expression of LAP2α, the unique LAP2 isoform that is not anchored at the INM, is essential for the nucleoplamsic localization of A-type lamins (Naetar and Foisner, 2009). Whereas lamin A/C localized at the nuclear periphery binds heterochromatin in a LAP2α-independent manner, the small fraction of A-type lamins localized in the nucleoplasm interacts with euchromatin in a LAP2α-dependent manner (Figure 1) (Dechat et al., 2000; Johnson et al., 2004; Kolb et al., 2011; Gesson et al., 2016). The knock-down of LAP2α resulted in the shift of lamin A/C binding toward more heterochromatic regions. Interestingly, the *LMNA* p.K32del mutation, responsible for severe muscle weakness and wasting, results in the unique localization of A-type lamins within the nucleoplasm in a LAP2α-independent fashion (Bertrand et al., 2012; Pilat et al., 2013). Likewise, the p.R388P A-type lamins mutant that is also uniquely observed in the nucleoplasm has been shown to have altered interaction with LAD (Paulsen et al., 2017).

In the nucleoplasm, lamin A/C has specific protein partners, including the retinoblastoma protein (Rb), a key regulator of cell proliferation (Figure 1) (Mancini et al., 1994; Novitch et al., 1996; Markiewicz et al., 2002; Pekovic et al., 2007). Studies using *Lmna*^Δ^^8−11/Δ8−11^ mouse MEF showed reduced levels of Rb when compared with controls, concluding that Rb-lamin A/C interactions are required for the stability, localization, and activity of Rb (Johnson et al., 2004) and showing that the localization of lamin A/C within the nucleoplasm is important for cell cycle regulation and cell differentiation (Cohen et al., 2013). Similarly, at the nuclear periphery, emerin binds directly to many transcription regulators, including Lmo7 (Holaska et al., 2006), ß-catenin (Markiewicz et al., 2006) and BAF (Holaska and Wilson, 2007). Lmo7 is a transcriptional factor involved in the activation of myogenic differentiation genes, such as *MyoD, Myf5*, and *Pax3* (Dedeic et al., 2011). Its binding to emerin at the nuclear periphery inhibits the binding and the transcriptional activity of Lmo7. A recent study showed that *Lmo7*-null mice present with a myopathic phenotype similar to that seen in other EDMD mouse models (Mull et al., 2015). Emerin also participates in the regulation of myogenic differentiation and skeletal muscle regeneration by preventing ß-catenin accumulation to the nucleus and the subsequent upregulation of ß-catenin-target genes (Markiewicz et al., 2006). A massive nuclear accumulation of ß-catenin was observed in fibroblasts from patients with X-EDMD, leading to an autostimulatory growth phenotype (Markiewicz et al., 2006).

EDMD proteins also established interactions with chromatin modifiers. Lamin A/C is required for the correct Polycomb group (PcG) protein nuclear compartmentalization (Cesarini et al., 2015). PcG proteins are epigenetic repressors that control numerous target genes during differentiation (Lanzuolo and Orlando, 2012). Polycomb repressive complex 2 (PCR2), one of the best characterized PcG protein complexes together with PCR1, has been shown to interact with lamin A/C in the nucleoplasm (Figure 1) (Cesarini et al., 2015) and its localization at the nuclear periphery is required for muscle differentiation (Wang et al., 2011). Lamin A/C knock-down leads to the dispersion of PcG proteins in the nucleus, causing an aberrant transcriptional reactivation of PcG targets and consequently leading to premature myogenesis (Cesarini et al., 2015). It has been also reported that FHL1 interacts with RING1, one of the components of PcG protein complex (Qin et al., 2004). FHL1 suppresses the RBP-J-mediated transcription by competing with transactivators for the binding motif RBP-J (Taniguchi et al., 1998) and by recruiting RING1 to RBP-J (Qin et al., 2004). The histone deacetylase HDAC3 binds to LAP2ß and emerin (Somech et al., 2005; Demmerle et al., 2012), the histone acetyltransferase hALP1 binds SUN1 (Chi et al., 2007) and LBR interacts with MeCP2, a DNA methylating enzyme (Guarda et al., 2009). Emerin directly interacts and regulates the activity of HDAC3, regulating indirectly the expression and nuclear localization of myogenic differentiation genes, such as *Myf5, MyoD* and *Pax7*. It has been shown that EDMD-causing emerin mutations are not able to bind to HDAC3 (Demmerle et al., 2012, 2013). Treatment of emerin-null cells with theophylline, an HDAC3-specific activator, improves myotube formation, whereas the addition of the HDAC3-specific inhibitor RGFP966 blocks myotube formation in wild-type and emerin-null myogenic progenitors (Collins et al., 2017), suggesting that the altered interaction between emerin and HDAC3 is important in the EDMD disease mechanism.

Lamins are also involved in DNA repair. A-type lamins stabilize 53BP1 protein levels and promote SIRT6-mediated DNA repair, preserving the integrity of the genome (Gonzalez-Suarez et al., 2011; Ghosh et al., 2015). Several *LMNA* mutations, including some leading to EDMD, have been shown to cause an upregulation of cysteine protease Cathepsin L (CTSL). This upregulation leads to the degradation of 53BP1 and changes in its cellular distribution, resulting in defective double strand breaks (DSB) DNA repair (Gonzalez-Suarez et al., 2011). Moreover, lamin A/C interacts with histones H2AX and its phosphorylated form (γH2AX) (induced by DNA damage), maintaining the positional stability of DNA damage repair foci. By contrast, in *Lmna*^Δ^^8−11^ mouse MEF the mobility of DNA repair foci is increased, evidencing the participation of A-type lamins in the spatial anchor of the genome (Mahen et al., 2013).

Thus, since the first mutation identified in 1994 in *EMD*, our knowledge of the nuclear envelope proteins functions and related pathomechanisms leading to EDMD and striated muscle laminopathies has tremendously expanded. Each new report confirms and reinforces the combination of the pathomechanistic hypotheses proposed by Hutchison and colleagues (Hutchison et al., 2001), i.e., the structural/mechanical and the gene regulation hypotheses.

## Future therapies

**Figure 2:**
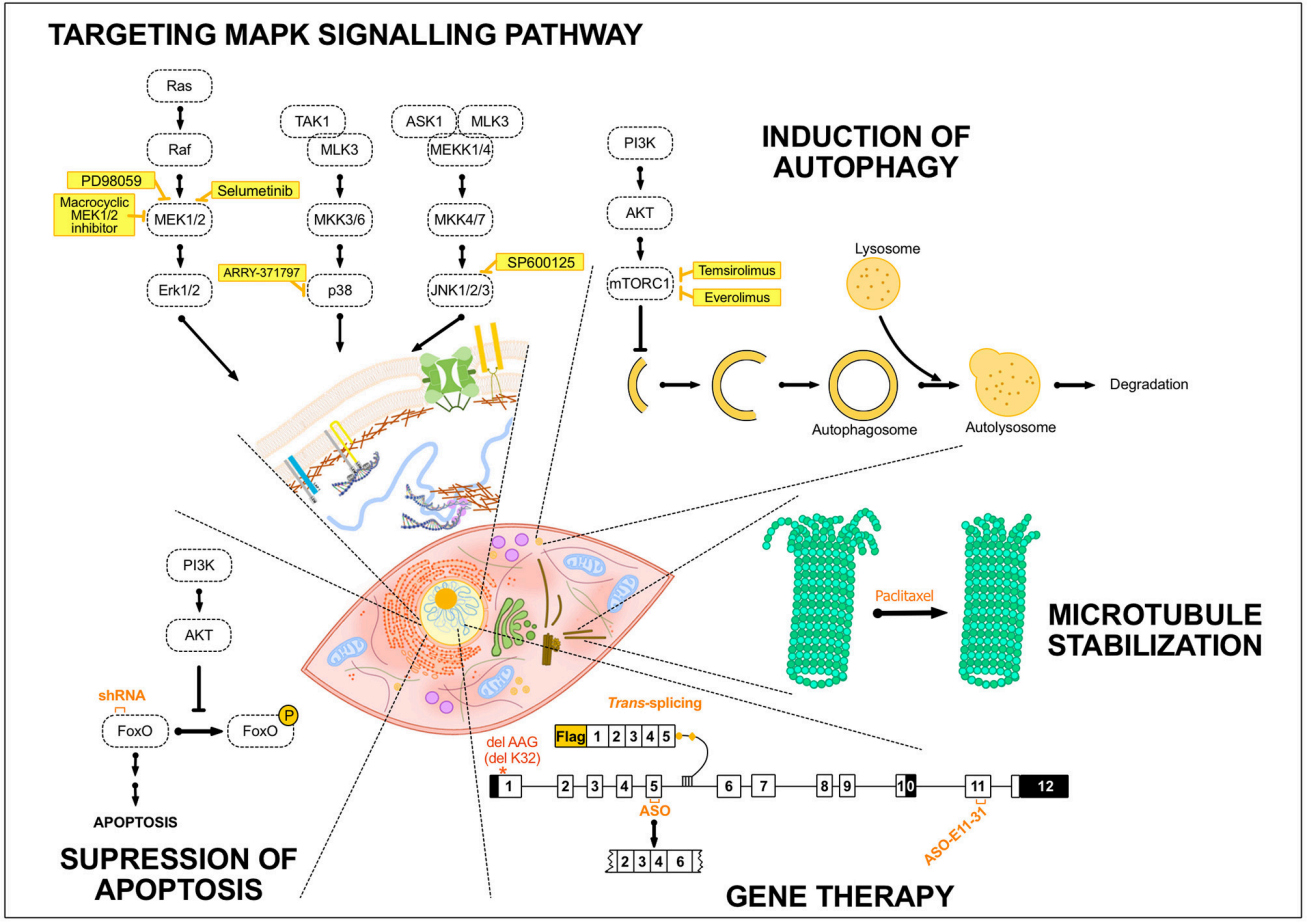
Summary of the potential treatments tested so far in EDMD. Schematic drawing representing several treatment strategies that have been developed for EDMD. The portrayed treatments target diverse mechanisms occurring either in the cytoplasm, e.g., autophagy in the lysosomes, or in the nucleus, including exon-skipping or trans-splicing strategies. The treatment strategies addressed to EDMD have generally been developed to target MAPK signaling pathway or to induce autophagy and these include different inhibitors of components of these pathways such as selumetinib or temsirolimus. To target apoptosis, the utilization of shRNA specific to Fox O1 and 3 has been developed. Others treatments and strategies such as the use of paclitaxel to stabilize microtubules or gene therapy to convert the mutant transcript into a normal transcript or remove an in-frame exon containing a mutation have been studied. AKT, protein kinase B; ASK1, apoptosis signal-regulating kinase 1; ASO, antisense oligonucleotide; ERK, extracellular signal-regulated kinase; FoxO, forkhead box O; MEK1/2, MAPK/ERK kinase 1/2; MEKK1/4, mitogen-activated protein kinase kinase kinase 1/4; MKK3/6, mitogen-activated protein kinase kinase 3/6; MLK3, mitogen-activated protein kinase kinase kinase 11; mTORC1, mammalian target of rapamycin complex 1; PI3K, Phosphoinositide 3-kinase; Raf, proto-oncogen serine/threonine-protein kinase; TAK1, mitogen-activated protein kinase kinase kinase 7.

No specific treatments exist for EDMD and related striated muscle laminopathies. Reports related to treatments and development of adapted specific therapies focused essentially on EDMD linked to *LMNA* mutations. Today, therapy is preventive and/or symptomatic. Supportive care, e.g., orthopedic procedures to minimize progression of joint contractures and spinal deformity, has been proposed to patients and in the most severe cases mechanical aids are required (Bonne and Quijano-Roy, 2013). Patients might also present respiratory insufficiency requiring nocturnal or continuous mechanical ventilation, especially in children with the congenital form (L-CMD, Table 1) or in early-onset EDMD patients (Quijano-Roy et al., 2008). All striated muscle laminopathies associated to *LMNA* mutation share a common denominator, the cardiac involvement, which represents the most life-threating feature of the pathology. Cardiac phenotype is mainly characterized by progressive conduction system defects, manifesting as sick sinus syndrome, atrioventricular block, or bundle branch blocks. Ventricular arrhythmia and systolic dysfunction are also common and all together often lead to impairment of myocardial function, development of heart failure, and cardiac transplant or premature death. Standard cardiac treatments including angiotensin II converting enzyme (ACE) inhibitors or angiotensin receptor blockers are widely used with treatment starting early (Lu et al., 2011). ACE converts angiotensin I into the active vasoconstrictor angiotensin II, indirectly increasing blood pressure and consequently increasing heart rate and vascular tone (Coates, 2003). Despite the use of conventional drugs for cardiovascular diseases (e.g. beta-blockers), heart failure almost always progresses; highlighting the urge to find new treatments for these disorders. Since mutations in *LMNA* gene were associated with laminopathies in 1999 (Bonne et al., 1999), numerous potential treatment options have emerged and been evaluated in mouse models (Table 2). The treatment strategies have generally been developed to target MAPK signaling pathway, to induce autophagy or to inhibit apoptosis but new therapies are rising (Figure 2). In this section, we review the different treatment strategies that have been studied so far.

### Targeting MAPK signaling pathway

MAPK signaling pathways are important transduction cascades initiated by extracellular mitogens, growth factors and cytokines at the cell surface and finalized to the nucleus to control gene expression (Davis, 1993). They regulate different cellular processes including cell proliferation and differentiation, survival, and apoptosis. The MAPK pathway is composed of four signaling cascades usually named according to the MAPK central component of each cascade: MAPK/ERK1/2 or classical pathway, JNK, p38 and ERK5 signaling cascades (Keshet and Seger, 2010). As mentioned earlier, Muchir and co-workers observed an activation of ERK, JNK and p38α cascades in hearts from *Lmna*^H222P/H222P^ mice (Table 2), linking the activation of MAPK pathways due to *LMNA* mutations to cardiomyopathy (Muchir et al., 2007b, 2012b). Since then, inhibitors of MAPK have become a promising therapeutic option in *LMNA* cardiomyopathy. Various inhibitors targeting different components of the cascade have been already developed and tested in cancer (Dudley et al., 1995; Zhao and Adjei, 2014; Maik-Rachline and Seger, 2016). Therefore, pharmacological inhibition of ERK1/2 was tested to prevent the cardiac dysfunction in a laminopathic mouse model (Figure 2). The allosteric inhibitor of MEK1/2 (PD098059) was one of the first MEK (the MAPK kinase that phosphorylates ERK1/2) inhibitors to be tested in these mice, delaying the development of left ventricular dilatation and improving function (Muchir et al., 2009). A more potent and selective inhibitor, i.e., Selumetinib (Figure 2) also lead to an improvement in left ventricular dilatation, an increase in cardiac fractional shortening, decreased cardiac fibrosis, as well as, a prolongation in survival compared to placebo-treated mice (Muchir et al., 2012a). More recently, a novel allosteric, macrocyclic MEK1/2 inhibitor was designed and evaluated in *Lmna*^H222P/H222P^ mice (Wu et al., 2017). This drug also resulted in an improvement of left ventricular systolic function, a decrease in left ventricular fibrosis, as well as, a beneficial effect on skeletal muscle structure and pathology and a prolonged survival.

Given that enhanced activities of JNK and p38α signaling were also observed in hearts from *Lmna*^H222P/H222P^ mice (Muchir et al., 2007b, 2012b), inhibitors of both JNK and p38α cascades were tested in *LMNA* cardiomyopathy (Figure 2). *Lmna*^H222P/H222P^ mice treated with SP600125, an inhibitor of JNK signaling, or with ARRY-371797, a p38α inhibitor, both showed a delay in the development of left ventricular dilatation and a reduction in the decrease of fractional shortening compared to placebo-treated mice (Wu et al., 2010; Muchir et al., 2012b). This led to the first clinical trial in patients with cardiomyopathy caused by *LMNA* mutations using ARRY-371797 (NCT02057341).

### Induction of autophagy

mTOR signaling pathway is involved in many fundamental cellular processes, from protein synthesis to autophagy (Figure 2). mTOR is a serine/threonine kinase that forms the core of two distinct protein complexes, mTORC1 and mTORC2. During cell growth, mTORC1 plays a central role in controlling production of proteins, lipids, and nucleotides as well as downregulating autophagy; mTORC2 also increases cellular proliferation and survival (Saxton and Sabatini, 2017). While mTORC1 is inhibited by rapamycin in an allosteric and specific manner, prolonged rapamycin treatment is needed to abrogate mTORC2 signaling (Sarbassov et al., 2006). Rapamycin and semi-synthetic rapamycin analogs with improved pharmacokinetics properties are widely used in cancer and some of them have reached various stages of clinical development, but only a few, e.g., temsirolimus and everolimus, have been approved by the FDA for their use in cancer treatment (Benjamin et al., 2011; Janku et al., 2018). Choi et al. demonstrated an activation of both AKT (activator of mTORC1) and mTORC1 as well as a defective autophagy in hearts of *Lmna*^H222P/H222P^ before the onset of clinically detectable cardiomyopathy. The treatment of 14 weeks old *Lmna*^H222P/H222P^ mice with temsirolimus reactivated autophagy and decreased left ventricular end-systolic dilatation, increased fractional shortening but did not prevent cardiac fibrosis in temsirolimus-treated mice compared to placebo-treated mice (Choi et al., 2012). Additionally, it has been recently published that everolimus is also improving the phenotype in fibroblast cell lines from six laminopathy patients with different *LMNA* mutations (DuBose et al., 2018). All these studies suggest that rapamycin-related mTORC1 inhibitors such as temsirolimus or everolimus, may be potential treatments for striated muscle laminopathies (Figure 2).

### Suppression of apoptosis

A recent study showed the activation of forkhead box O transcription factors (FoxO TFs) 1 and 3 and a deleterious role for overactivation of FoxO TFs in the hearts of *Lmna*^Δ^^8−11^ mice and cardiac myocytes (Auguste et al., 2018). By contrast, no significant differences in phosphorylated FoxO3a were previously observed in hearts of *Lmna*^H222P/H222P^ mice compared to control mice (Choi et al., 2012). FoxO TFs are downstream targets of AKT. AKT phosphorylates FoxO TFs inhibiting their function and contributing to cell survival, growth and proliferation (Zhang et al., 2011) (Figure 2). Auguste et al. showed that subcutaneous injection of AAV9 encoding shRNA specific to FoxO1 and 3 in *Lmna*^Δ8−11^ newborn mice nonetheless efficiently reduced cardiac FoxO1 and 3 transcripts to wild-type levels but also reduces transcripts levels of FoxO target genes involved in apoptosis. In addition, knockdown of FoxO TFs in the heart resulted on a ~2-fold prolongation of survival in the *Lmna*^Δ8−11^ mice compared to untreated *Lmna*^Δ8−11^ mice (Auguste et al., 2018). These findings identify another potential target to prevent or ameliorate cardiac phenotype in laminopathies.

### Gene therapy

It has been largely described in the past that mutant *LMNA* transcripts have a dominant-negative effect (Azibani et al., 2014). Therefore, the conversion of the mutant transcript into a normal transcript or the removal of an in-frame exon containing a mutation could potentially improve pathogenic phenotypes in laminopathies (Figure 2). Scharner et al. demonstrated that the use of antisense oligonucleotides to skip *LMNA* exon 5 in human resulted in truncated human Lamin A or C that localized normally, restoring both nuclear shape and endogenous Lamin B1 and emerin localization (Scharner et al., 2015). Additionally, skipping of exon 11 might be another approach to treat other laminopathies caused by missense mutations in exon 11. Lee JM et al. used an exon 11 antisense oligonucleotide (ASO E11-31) to shift the balance between lamin C and prelamin A splicing thus, increasing lamin C production in mouse and human fibroblasts, as well as reducing the expression of progerin in fibroblasts derived from patients with Hutchinson-Gilford progeria syndrome (HGPS) (Lee et al., 2016). Finally, we have recently provided the first evidence of reprogramming *LMNA* mRNA *in vitro* by spliceosome-mediated RNA trans-splicing (SMART) as a therapeutic approach for striated muscle laminopathies (Azibani et al., 2018) (Figure 2). This technology allows the incorporation WT exons and the concomitant deletion of the corresponding mutated exons during the step of pre-mRNA maturation via transsplicing. Using this approach in primary myoblasts from *Lmna*^Δ^^K32/Δ*K*32^ mice, we were able to rescue part of the nuclear abnormalities, increasing the proportion of lamin A/C at nuclear periphery, as well as, both emerin and lamin B1 mislocalization. However, we only detected an extremely modest increase in lamin A/C mRNA expression after systemic injection in pups, probably due to the low *trans*-splicing rate.

### Alternative therapeutic approaches

Other therapeutic options have been investigated. Arimura et al. showed that the treatment with pyridazinone derivative calcium (Ca^2+^) sensitizing agent SCH00013 in *Lmna*^H222P/H222P^ mice resulted in an amelioration of systolic dysfunction, a prolongation in life expectancy, a reduction in cardiac interstitial fibrosis and a modulation of the expression of genes involved in cardiac remodeling (Arimura et al., 2010). The mechanisms for the beneficial effect of SCH00013 have not been elucidated yet and more studies are needed but these findings suggest Ca^2+^ sensitizer as an option for *LMNA* cardiomyopathy treatment.

Another approach specifically targets *LMNA* mutations leading to premature stop codon. Lee et al. showed that treatment of cardiomyocytes derived from human induced pluripotent stem cells carrying different *LMNA* mutations with PTC124, a pharmaceutical drug that selectively induces translational ribosomal read-through over the premature stop codon, increased the production of full-length lamin A/C proteins although only in one of the patient's cell line (Lee et al., 2017). It has been already tested in a phase 3 trial for Duchenne muscular dystrophy (McDonald et al., 2017), therefore this may be an alternative option for the treatment of nonsense-mutation causing laminopathies.

As mentioned before, we showed an altered distribution of Cx43 in *Lmna*^H222P/H222P^ mice due to microtubule cytoskeleton alteration and decreased acetylation of α-tubulin, leading to electrical conduction disturbances (Macquart et al., 2018). Paclitaxel treatment, a drug used in therapeutics for several forms of cancer and already tested in patients underlying cardiac dysfunction (Gollerkeri et al., 2001), increased the acetylated form of α-tubulin in *Lmna*^H222P/H222P^ treated mice, as well as relocalized Cx43 at the intercalated disks (Macquart et al., 2018). These results revealed Paclitaxel as a potential candidate treatment to stabilize microtubule cytoskeleton in EDMD patients.

Finally, another potential treatment in laminopathies arise from the DNA repair defects observed upon loss of A-type lamins (Gonzalez-Suarez et al., 2011). Gonzalez-Suarez and colleagues showed that the inhibition of CTSL by vitamin D treatment resulted in the stabilization of 53BPA and the induction of DNA DSBs repair.

## Conclusions

The identification of new proteins causing EDMD and EDMD-like myopathies has provided new insights into the role of nuclear envelope proteins in the cell. The use of animal models has permitted the identification of signaling pathways that are deregulated when these proteins are absent or mutated, helping to understand the mechanisms involved in these pathologies. Therapeutic treatments modulating these pathways and new gene therapy approaches have shown some benefits in mice. However, further studies are needed to better understand how these altered mechanisms lead to these pathologies and how they lead to such different phenotypes.

## Author contributions

AsB and BMR original writing and edition of the manuscript. AM edition of the manuscript. GB review and edition of the final manuscript. AnB supervision, writing, and edition of the manuscript.

### Conflict of interest statement

BMR is employed by Sanofi, a global biopharmaceutical company focused on human health. The remaining authors declare that the research was conducted in the absence of any commercial or financial relationships that could be construed as a potential conflict of interest.
